# New ways of working; delivering better care for people with long-term conditions*

**DOI:** 10.1080/17571472.2017.1361619

**Published:** 2017-08-11

**Authors:** Victoria Tzortziou Brown, Irem Patel, Nicola Thomas, James Tomlinson, Rachel Roberts, Hugh Rayner, Neil Ashman, Sally Hull

**Affiliations:** a Centre for Primary Care and Public Health, Barts and the London, School of Medicine and Dentistry, London, UK; b King's College London School of Medicine, King’s College Hospital NHS Foundation Trust, London, UK; c School of Health and Social Care, London South Bank University, London, UK; d Renal Medicine, Faculty of Medicine, Imperial College London, Institute of Clinical Sciences (ICS), London, UK; e Primary Care Education and Development, HEE North Central and East London, London, UK; f Department of Renal Medicine, Birmingham Heartlands Hospital, Birmingham, UK; g Renal Medicine, Barts Health NHS Trust, The Royal London Hospital, London, UK

**Keywords:** Outpatient, transformation, long term conditions, general practice, CKD, COPD, primary care

## Abstract

**Background:**

The cost-effectiveness of the traditional outpatient model for specialist care provision is increasingly being questioned in view of the changing patient needs, workforce challenges and technological advances.

**Setting:**

This report summarises two RCGP London events showcasing new ways of delivering care for long-term conditions.

**Questions:**

What are the alternative approaches to the traditional outpatient model and do they have common themes? What are the challenges and opportunities of these new models of care?

**Methods:**

Presentation of examples of new ways of long-term condition care delivery and round-table facilitative discussion and reflection on the challenges and solutions around service re-design and implementation, the commissioning and funding of new models of care, the facilitation of system-wide learning and the collection of data for evaluation.

**Results:**

Different ways of delivering care for people with Chronic Kidney Disease (CKD) and Chronic Obstructive Pulmonary Disease (COPD) were presented. Most of the interventions included virtual clinics (during which patient care was reviewed by a specialist remotely without the need for a face-to-face consultation), improved communication between primary and secondary care clinicians, an element of referral triage/prioritisation, the use of trigger tools to identify people at risk of deterioration, patient education and a multi-disciplinary approach.

**Discussion-conclusions:**

Different models to the traditional outpatient long-term condition care are feasible and can result in improvements in the quality of care and staff satisfaction. However, such initiatives require careful planning, close collaboration between health care professionals and allocation of appropriate resources and training within primary care. There is also a need for systematic evaluation of such pilots to assess their cost-effectiveness and their acceptability to clinicians and patients. This requires systematic collection of population level data, agreement on the key outcomes for evaluation and a commitment of all stakeholders to sharing learning and resources to enable continuous improvement.

## Why this matters to me

Demand for health care is rising as people live longer, have more complex health problems and more advanced treatments become available. Health care professionals are overworked and treatment waiting times are becoming longer. At the same time, the NHS faces its greatest financial challenge of recent times. The model of care provision has remained relatively unchanged for many decades despite technological advances and patient pressure for more person-centred care. Local innovative solutions are often developed and piloted but these are rarely evaluated and disseminated and therefore learning is not always shared.

The two events presented here aimed to bring together health care innovators to share their new approaches to long-term condition care provision and learn from each other. These new ways of working across primary and secondary care and the resultant shared learning will hopefully inspire others.

## Key message

By working together we can improve care for people with long-term conditions.

## Background

The traditional model for accessing non-urgent specialist advice and care within the NHS is via face-to-face consultations within outpatient hospital clinics. The way of delivering such outpatient services has remained relatively static for several decades. However, in view of the changing demographics, rising clinical need and workforce challenges, waiting times for outpatient appointments and specialist treatment are breaching the 18-weeks targets [1] and outpatient costs are escalating [2]. In addition, patients` appeals to be put in charge of their care [3] are not being prioritised and technological advances such as the electronic patient records are not being fully utilised. There is therefore a need for a review of the current system and consideration of the alternatives.

Different models of accessing specialist advice and care include the partial substitution of hospital clinicians with primary care clinicians (such as GPs with special interests, nurse and extended scope practitioners), the relocation of hospital specialists to the community or virtual setting (e.g. attachment to primary care teams, ‘virtual’ clinics, telemedicine) and joint working between specialists and primary care practitioners via shared care arrangements and consultation liaison [4].

Outpatient transformation has been prioritised by commissioners and providers in many areas across the UK and alternative ways of delivering outpatient services are increasingly being explored and piloted [5–10]. The development of such new approaches is particularly important in the area of long-term condition (LTC) management where demand is rising due to an aging population and multi-morbidity [11]. It is estimated that 64% of outpatient appointments and around 70% of the total health and care spend in England (£7 out of every £10) is attributed to caring for people with LTCs [11]. Apart from being costly, outpatient hospital care is disease-focused rather than person-focused and therefore is often not the ideal setting for managing multi-morbidity and complexity and adopting person-centred care planning approaches [12].

There are a few examples of innovative approaches to LTC management across the UK but limited published evidence on the effectiveness, cost-effectiveness and acceptability of these new models of care. [13–15] Several of these new approaches have been developed around community-wide Chronic Kidney Disease (CKD) management programmes.

CKD is defined as either kidney damage or glomerular filtration rate (GFR) less than 60 mL/min/1.73 m^2^, or both, persisting for at least 3 months [16]. The National CKD audit across England and Wales estimated a CKD prevalence of approximately 5.5% with the majority of people being at stage 3 which is usually asymptomatic [17]. Only 2% of patients with CKD will progress to end-stage renal disease requiring highly specialised renal replacement therapy [16].

Therefore, the bulk of CKD management can take place in a GP setting which allows a more holistic approach. This is important because CKD often co-exists with other conditions such as hypertension, cardiovascular disease and diabetes while obesity and smoking are associated risk factors. [16,18–21] Promoting a healthy lifestyle, smoking cessation and weight loss as well as encouraging better control of diabetes and blood pressure are advocated as part of good clinical practice [16]. Careful review of both prescribed and over-the-counter medication is also paramount in view of the fact that the use of nephrotoxic drugs (including non-steroidal anti-inflammatory drugs) has been recognised as a major cause of iatrogenic renal disease [22]. It can be argued that general practice with its biopsychosocial approach, continuity and accessibility offers a more appropriate and less costly clinical setting than outpatient clinics for such holistic reviews. General practice working within extended, community teams is better placed to provide coordinated care for multi-morbidities by aligning care pathways of all LTCs and developing locally-based care plans and self-care promotion [12]. The above and increasing concerns of over-diagnosis and over-medicalisation [23] may explain the recent emphasis of shifting CKD care out of hospital and into the community.

However, despite the increasing number of innovative projects within CKD management, there has not been a documented attempt to describe these, encourage professional connections and support shared learning. Similar initiatives have been developed nationally for other common LTCs such as diabetes and COPD. It is unclear whether there are common principles and learning from these which could be applied more broadly in new models of care.

## Aims and objectives

### Renal symposium

The aim of the renal symposium was to bring together front-line clinicians and renal experts to reflect on innovative CKD pathways, including their implementation and evaluation.

### City Health Conference workshop

The workshop that followed the renal symposium aimed to disseminate the learning on renal outpatient transformation, present alternative approaches for the management of a different long-term condition (COPD), explore the similarities and differences of these different ways of delivering care and identify common themes.

## Objectives

(a) Share knowledge and best practice on innovative approaches to LTC service provision;
(b) Gain insight into clinicians` understanding and attitudes towards new ways of working;
(c) Provide an opportunity for collaboration and dissemination of learning and good practice.

## The events

### Renal symposium-31st January 2017

The renal symposium presented innovations in renal care in five NHS trusts: Barts Health NHS Trust, Imperial College NHS Trust, Royal Free NHS Foundation Trust, Heart of England NHS Foundation Trust and Epsom and St Helier University Hospital NHS Trust.

Following the presentations by the five NHS trusts, there was a **round table discussion** which offered an opportunity for reflection on the presentations and discussion on issues around implementation, funding, clinician and patient education and evaluation.

### City Health Conference workshop-30th March 2017

The second event was hosted as part of the RCGP London City Health Conference 2017. It presented new ways of delivering renal and COPD care.

It also gave an overview of developments and opportunities around **inter-professional learning.**

Participants in both events included a wide range of professionals (General Practitioners, secondary care consultants, primary and secondary care nurses, allied health care professionals, commissioners and NHS managers).

## Different ways of delivering **LTC** care

Table 1 summarises the different approaches to delivering CKD and COPD care as these were presented in the two events. Most of the interventions included virtual clinics (during which patient care was reviewed by a specialist remotely without the need for a face-to-face consultation), good communication between primary and secondary care clinicians, an element of triage/prioritisation, the use of trigger tools, GP and patient education and a multi-disciplinary approach (involving doctors, nurses, managers and commissioners).

**Table 1.** Innovative care models for the delivery of CKD and COPD management.

NHS trust	Intervention and results
Renal care	Intervention
Barts Health NHS Trust & Clinical Effectiveness Group (CEG) model	*E-Clinic*: Community-based nephrologist doing e-clinic in EMIS Web. All referrals electronic (through e-referral), all reviews and opinions recorded in EMIS Web, locally agreed guidelines.
Location: Four CCGs in East London (Tower Hamlets, City & Hackney, Newham, Waltham Forest)	
	*Shared records*
	*Education of primary care clinicians*: Practice based education
	*Patient education*: Patient 1:1 and group education
	*Community CKD overview*: CCG/Practice dashboards with targets, CKD prevalence searches to identify patients, ‘Trigger tools’ to alert GPs to patients with a falling e-GFR
	Results
	70% of referrals are now managed without the need for patients to attend a hospital appointment.
	During 2015 there was a rapid reduction in the wait time for a specialist appointment.
	The trigger tool supports practice reflection on falling eGFR results, with high risk cases being referred for renal review.
Renal care	Intervention
Imperial College Healthcare NHS Trust	*E-clinic*: Use of electronic referral forms compatible with Systm1 and EMIS, local guidelines based on NICE guidance
Location: Joint initiative between 8 local CCGs and the Trust	*Education of primary care clinicians*: funded nurse focusing on education
	*Planned discharge* from secondary care, discharge information both to patient and GP, management plan in place including criteria for re-referral.
	Results
	Full engagement across the 8 NW London CCGs
	>30 community education sessions
	>300 patients transferred from renal outpatients into shared care
	Multidisciplinary educational materials and guidelines agreed in relation to diabetes and also Heart Failure and CKD – discussions beginning in relation to the frail elderly
	Active e-advice service running approximately 6 emails per week – 75% prevent referral
Renal care	Intervention
Royal Free NHS Foundation Trust	*E-clinic*: triage, clear referral criteria
	*EMIS record sharing* agreement
	*Nurse led clinics* and joint CKD-diabetes nurse led clinics
	*Patient education*
	*Care planning*
	Preliminary results
	High reported patient satisfaction rates with clinics and improved knowledge
Renal care	Intervention
Heart of England NHS Foundation Trust (ASSIST CKD programme) [24]	*Trigger tool*-Laboratory surveillance and selective graphical reports: Identifying patients at high risk (i.e. deteriorating eGFR)
	*Education of primary care clinicians*
	*Patient education and care planning*: Letters to patients in a way they can understand, giving them their results and plan of action
	Results
	1600 graphs reported to GPs per year
	Lowest rate of late presentation for chronic dialysis
	Highest % rate of early presentation for chronic dialysis
	Reduction in need for renal replacement therapy (RRT)
Renal care	Intervention
Epsom and St Helier University Hospitals NHS Trust (part of ASSIST-CKD programme)	*Trigger tool*: First lab to fully automate the process within the laboratory computer system. Other labs log onto a separate system to produce results once a week. First lab to generate reports and send them to the GP electronically (overnight). Other labs print and post graphs
	Results
	160 graphs reviewed per week
	42 graphs sent out to GPs per week overall (~90 practices)
	Number of graphs sent per week has fallen from early days of 65 to a more stable 30 per week (31 vs 23% of graphs viewed)
COPD care	Intervention
King’s Health Partners, Southwark and Lambeth CCGs	*E-clinic*: virtual clinics in primary care (face to face clinical sessions between primary and specialist clinicians)
	*Shared respiratory formulary*
	*Shared records*
	*Education of primary care clinicians*
	*Admission avoidance and Early Supported Discharge* for COPD with care planning
	*Home Oxygen Assessment and Review*
	*7 day integrated respiratory team “without walls”*
	Results
	Significant shift in prescribing practice to reduce inhaler-related harm, waste and costs
	Reduction in high dose inhaled steroid prescribing with cumulative savings of £350,000 over first 7 quarters
	50% increase in pulmonary rehabilitation referrals from primary care
	Total COPD admissions reduced by 8%, uncomplicated COPD admissions reduced by 34%, length of stay reduced by 17%
	High rates of clinician and patient satisfaction

## Round table discussion-renal symposium

The round table discussion during the renal symposium covered four areas: the challenges and solutions around service re-design and implementation, the commissioning and funding of new models of care, the facilitation of system-wide learning and the collection of data and evaluation. Table 2 summarises the key points of these discussions.

**Table 2.** “New ways of working – challenges and solutions” – key points raised during the round-table discussion.

Implementation
How to gain “buy-in” from all stakeholders?
How to refer patients, share data and communicate between primary and secondary care?
*Challenges*
• Numerous and different IT systems currently utilised between practices and secondary care • IT needs to facilitate data exchange between primary and secondary care IT systems and support data collection on clinical outcomes and not just activity • There is often variable degree of engagement of different stakeholders; it is important to try to engage with as many as possible
*Solutions*
• Work and think as one system across primary and secondary care. Agree direction of travel/vision and identify champions at all levels (CCG, acute trust, general practice) • Review, manage & control access into the acute service using a single point of access • Promote the use of one shared care record across Long Term Condition management • Are GPs aware of e-advice service? Direct referrals into email before outpatients? • Use mutually agreed proformas/local guidance • Agree appropriate investigations and utilise triage and nurse assessments before a consultant outpatient appointment • Use similar alerts across labs & GP software systems (e.g. EMIS, SystmOne) • Support STP system-wide IT strategy
Commissioning and funding
How to fund, resource and sustain new ways of working?
*Challenges*
• Current system of Payment By Results (PBR) can be a barrier; it incentivises activity which may not be needed • The PBR system focuses solely on hospital funding; we need to consider ways to evaluate and fund the whole system, including general practice, especially if more work will be pushed towards general practice/community in the future
*Solutions*
• A block/capitated type of contract can give a sense of security to staff/provider and allow the development of new ways of working • Allocate funding to IT infrastructure to ensure connections between GP and hospital IT systems, invest in intelligent data sources such as pathology labs which merge data so that this can be interpreted easily over time and inform clinical practice • Important to invest on QI at practice/hospital levels and work with local academics (CEG model) to facilitate reflection and learning • Having a more flexible way of allocating funding, would also allow better involvement of other professionals such as nurses • Systems under financial pressure: this leads to short-term planning. Changes may need longer to be established and refined
System-wide learning
How to ensure all parties see and understand data?
*Challenges*
• Deficits in staff education: primary and secondary care clinicians do not always have a good understanding of each other’s roles and perspectives • Clinical letters not always easy for patients to understand
*Solutions*
• Benefits of more joint training days. • Develop opportunities of primary care exposure for specialty trainees and nurses • Educational resources need to be shared • Data-sharing: there is a need for clarity on data sharing in order to allow clinicians and patients to make informed decisions • Patient education: Such resources need to culturally appropriate and easy to understand • Use patient-focused letters instead of standard outpatient letters • Graphs for e-GFR are incredibly useful • Opportunities with STP development to communicate better, share learning and achieve large-scale changes
Data and evaluation
How to monitor progress?
*Challenges*
• Need agreement on minimum standard of quantitative and qualitative data Solutions • Important to have access to clinical outcomes and activity data at population level in order to evaluate the effectiveness and cost-effectiveness of new ways of delivering services – engage local academics, public health, IT leads • Important to agree on common outcomes to measure and evaluate. Some of these outcomes will be disease-specific but others will need to reflect quality of life, function and patient experience

IT = Information Technology; STP = Sustainability and Transformation Plan; PBR = Payment By Results; CEG = Clinical Effectiveness Group.

## Inter-professional learning-City Health Conference

The City Health Conference event included a presentation on how training has started to evolve in response to the changing population needs. Increasingly learners are seen as the vehicle for change and inter-professional learning is being promoted as the solution to improving teamwork and ensuring safety in an environment of rising medical complexity [25].

New care models can only be implemented if the workforce model of delivering care changes too. Education and training are key enablers to supporting these changes. Whilst current senior staff have been trained to deliver a model of care pretty much unchanged since the inception of the NHS it is vital that trainees coming through the current system are given the opportunity to think differently about care delivery and to adapt to and indeed lead the changes described earlier. The Five Year Forward View section on workforce training [26] warns ‘the risk is that the NHS will lock itself into outdated models of delivery unless we radically alter the way in which we plan and train our workforce’ echoed by the Royal College of Physicians Review of Integrated Care Training that states ‘Changes across medical education are required to equip the future workforce to deliver integrated care … all the contributors to this report have stressed the need for greater shared training’ [27].

In response to the above Health Education England (HEE) in North Central and East London developed initiatives of inter-professional learning and education including trainee rotations bridging primary and secondary care, multi-professional educational grand rounds and simulation workshops and Community Educator Provider Networks (CEPNs). HEE also launched a number of pilots focusing on General Practice trainees, attempting to address the core issues of working across organisational boundaries in order to improve collaboration and integration of clinical services and to encourage and equip clinicians to lead this programme of new ways of working. These pilots are presented in Table 3. A formal evaluation of these training programmes has now been commissioned by HEE in order to establish the value to trainees in preparing them for integrated care models and leadership, the benefits for patients and the cost effectiveness of such innovations.

**Table 3.** North Central and East London HEE education and training pilots.

Pilot schemes	Details
Exposure to primary and secondary care within the same week	This pilot involves training posts that are job planned to have time in both secondary and primary care within each week so that the trainee acquires a holistic perspective of the service. Such posts combine, for example, roles within the hospital Emergency Department and a primary care Urgent Care Centre, community paediatrics with paediatric A&E, or general practice gynaecology and specialist community gynaecology clinic. These sets of rotations are further supported by a 3-year quality improvement support package where trainees are introduced to the methodology of QI and are supported to deliver a clinical pathway improvement project across primary and secondary care
Musculoskeletal pilot	This pilot in musculoskeletal (MSK) services enables secondary care rheumatology trainees to sit side by side with GP trainees to see patients with complex MSK problems within a primary care setting and to learn from the mutual experience. Whilst initially the learning was conceived as transfer of specialist to generalist knowledge it soon became clear that specialty trainees had no understanding of the broad range of presentations encountered in primary care or the challenges faced in those circumstances. Sharing that experience has led to suggested improvements in the primary care management pathway for MSK patients
Care home pilot	This pilot has taken place in the Care Home setting where under the supervision of a GP, specialty trainees in General Practice, Old Age Psychiatry and Geriatric Medicine working with Community Pharmacists have carried through annual reviews of care home residents ensuring a holistic approach which included medicines rationalization, with subsequent reduction in potential morbidity and cost savings in drug costs, and individualized advanced care planning. Feedback from specialty trainees has been excellent and care home staff have felt much better supported in the management of a group of patients at high risk of hospital admission. The positive views about this programme have led to its adoption in North Central London

## Discussion

Both the renal symposium and the City Health Conference workshop showcased examples of new ways of delivering care for patients with LTCs which move away from the traditional and rather inflexible outpatient appointment system towards a more local, population-focused, integrated care approach.

Such a shift requires careful planning, close collaboration between primary and secondary care professionals and allocation of appropriate resources and training for General Practice and community staff [26]. Despite the existence of guidelines such as the NICE guidance on CKD and COPD management, the fact that both renal and lung function decline with age [27,28] means that increasingly a larger part of our population is affected by these conditions. The frequent co-existence of other LTCs, the multitude of factors that affect the rate of clinical deterioration and the poorly understood interaction of these risk factors with age [29] pose clinical challenges for primary care clinicians.

Therefore, close collaboration between experts in the field and front-line primary care clinicians is required so that any shifts of care can take place safely and result in cost-effective and sustainable improvements in the quality of services for patients. The divide between primary and secondary care is increasingly being bridged with primary care professionals taking on extended roles within specialised settings and secondary care specialists participating in joint community clinics [30]. Such approaches clearly require design around the needs of the local population but also necessitate longer-term consideration of training needs and reallocation of funding. Working in inter-professional teams is important for addressing the increasingly complex and challenging needs of an aging population as it allows sharing of expertise and perspectives and alignment of resources around the patient [31,32]. Inter-professional working and learning was a common component of all the interventions presented in our events.

Other common elements included virtual clinics, triage of referrals, good communication between primary and secondary care, health care professional and patient education, an MDT approach (involving doctors, nurses, managers, commissioners), the use of local guidelines and proformas and trigger tools to identify patients at clinical risk. Given each team`s experience represented different stages of development, many resources could be shared rather than trying to “reinvent the wheel”. There is a need for a central repository for patient leaflets, proformas and business cases, for example. Also, there is a need to communicate better with colleagues from other trusts and share learning either via virtual platforms or via similar symposia in the future.

Person-centredness, incorporating a tailored, holistic and more flexible approach to care with a focus on patient education and empowerment was also a component of most approaches presented and is in line with what patients themselves have called for, according to National Voices [3]. People have asked to be supported to understand their condition and be fully involved in decisions that affect their health, care and treatment. Patient educational initiatives were well-received in many of the regions adopting this approach (Table 1). Patient letters need to reflect this shift towards empowering the person with the condition (and their families and carers) avoiding medical jargon and acronyms. The “fully engaged scenario” is not only right in principle but also is paramount for the financial sustainability of our health and care system as highlighted in the Wanless report [33,34].

Better integrated and co-ordinated models of care that put the person at the centre, vertical integration and digitally enabled transformation have been recognised as important and are heavily promoted as a priority by NHS England [35] and health think tanks such as the Kings Fund [36]. Our two events showcased examples of such approaches to care and provided an opportunity for health-care staff to reflect on the benefits as well as the potential risks and barriers to working differently. Despite the increasing number of similar pilots across the country and the preliminary positive results in terms of patient experience and clinical outcomes, there is limited published evidence on the acceptability to clinicians and patients and an urgent need for evaluation of clinical benefits and cost effectiveness of such schemes. This requires systematic collection of population-level data, agreement on key outcomes and a commitment of all stakeholders to sharing learning and resources to enable continuous improvement.

## Speakers and contributors

Dr. Neil Ashman, Consultant Nephrologist, Barts Health NHS Trust.

Dr. Andrew Frankel, Consultant Nephrologist, Imperial College NHS Trust.

Sec Hoong, Senior Project Manager, Barts Health NHS trust.

Dr. Sally Hull, GP academic, Clinical Effectiveness Group, QMUL.

Sheila Johnston, Lead Nurse Chronic Kidney Disease, Royal Free NHS Foundation Trust.

Marta Lapsley, Consultant Chemical Pathologist, Epsom and St Helier NHS Trust.

Dr. Irem Patel, Consultant Respiratory Physician, Integrated Care, King’s Health Partners.

Helen Rainey, Nurse Chronic Kidney Disease, Barts Health NHS Trust.

Dr. Hugh Rayner, Consultant Nephrologist, Heart of England NHS Foundation Trust.

Dr. Rachel Roberts, Head of Primary Care Education and Development HEE North Central and East London.

Professor Nicola Thomas, Professor of Kidney Care, School of Health and Social Care, London South Bank University.

Dr. James Tomlinson, Nephrologist, Imperial College NHS Trust.

Dr. Victoria Tzortziou Brown, GP researcher and commissioner and Chair RCGP London Faculties.

Professor Robert Walton, Professor of Primary Care, Centre for Primary Care and Public Health, Blizard Insititute, QMUL.

## Funding

This work and related events were supported by NIHR (through the NIHR Clinical Lecturer symposium scheme of the Centre for Primary Care and Public Health, Barts and The London School of Medicine and Dentistry).

## Disclosure statement

No potential conflict of interest was reported by the authors.

## References

[1] Statistical Press Notice NHS referral to treatment (RTT) waiting times data March 2017. *NHS England**.* [Online] 2017 May 11 [cited 2017 May 15]. Available from: https://www.england.nhs.uk/statistics/wp-content/uploads/sites/2/2016/06/Mar-17-RTT-SPN-publication-version.pdf

[2] NHS England. *NHS inpatient elective admission events and outpatient referrals and attendances: Quarter Ending December 2016**.* [Online] 2017 [cited 2017 May 15]. Available from: https://www.england.nhs.uk/statistics/wp-content/uploads/sites/2/2013/04/QAR-commentary-Q3-1617-08675.pdf

[3] A Narrative for Person-Centred Coordinated Care. *National Voices**.* [Online]. [cited 2017 May 18]. Available from: https://www.nationalvoices.org.uk/sites/default/files/public/publications/narrative-for-person-centred-coordinated-care.pdf

[4] Roland M, McDonald R, Sibbald B Outpatient Services and Primary Care: A scoping review of research into strategies for improving outpatient effectiveness and efficiency. *National Primary Care Research and Development Centre**.* [Online] 2006 March [cited 2017 May 15]. Available from: https://www.netscc.ac.uk/hsdr/files/project/SDO_FR_08-1518-082_V01.pdf

[5] Outpatient Transformation Programme - Best in Class. *Wales NHS Health Board**.* [Online]. [cited 2017 May 15]. Available from: www.wales.nhs.uk/sitesplus/866/opendoc/218561

[6] Transforming Outpatient Services: Adopt Advice Only, Referral Feedback response and develop Clinical Dialogue. *NHS Scotland**.* [Online]. [cited 2017 May 15]. Available from: www.qihub.scot.nhs.uk/media/809081/adviceonly.pdf

[7] The Modern Outpatient: A Collaborative Approach 2017–2020. *NHS Scotland**.* [Online]. [cited 2017 May 15]. Available from: https://www.rcophth.ac.uk/wp-content/uploads/2017/02/23_NHSSCOTLAND_the-modern-patient-a-collaborative-approach.pdf

[8] Hunter J, Claridge A, James S, et al Improving outpatient services: the Southampton IBD virtual clinic. Postgrad Med J. 2012;88:487–491. 10.1136/postgradmedj-2012-100123rep 22822228

[9] Chua R, Craig J, Wootton R, et al Randomised controlled trial of telemedicine for new neurological outpatient referrals. J Neurol Neurosurg Psychiatry. 2001;71:63–66. 10.1136/jnnp.71.1.63 11413265 PMC1737484

[10] Rogers H, Maher L, Plsek PE New design rules for driving innovation in access to secondary care in the NHS. BMJ. 2008;337:a2321 10.1136/bmj.a2321 19074942

[11] DH/Long Term Conditions. Long Term Conditions Compendium of Information: Third Edition. *Department of Health**.* [Online] 2012 May 30 [cited 2012 May 15]. Available from: https://www.gov.uk/government/uploads/system/uploads/attachment_data/file/216528/dh_134486.pdf

[12] Unadkat Neha, Evans Liz, Nasir Laura, et al Taking diabetes services out of hospital into the community. London J Prim Care. 2013;5:87–91. doi:10.1080/17571472.2013.11493386. PMC4413714 25949676

[13] Greenhalgh T, Vijayaraghavan S, Wherton J, et al Virtual online consultations: advantages and limitations (VOCAL) study. BMJ Open. 2016;6:e009388. doi:10.1136/bmjopen-2015-009388 PMC4735312 26826147

[14] Kelly JJ, Sweigard KW, Shields K, et al Safety, effectiveness, and efficiency: a web-based virtual anticoagulation clinic. Jt Comm J Qual Patient Saf. 2003;29:646–651. 10.1016/S1549-3741(03)29076-6 14679867

[15] Pinnock H, Hanley J, McCloughan L, et al Effectiveness of telemonitoring integrated into existing clinical services on hospital admission for exacerbation of chronic obstructive pulmonary disease: researcher blind, multicentre, randomised controlled trial. BMJ. 2013;347:f6070 10.1136/bmj.f6070 24136634 PMC3805483

[16] National Institute for Health and Care Excellence. NICE guideline: Chronic kidney disease in adults: assessment and management. *NICE guidelines**.* [Online] 2014 July [cited 2017 May 8]. Available from: https://www.nice.org.uk/guidance/cg182 32208570

[17] Hill NR, Fatoba ST, Oke JL, et al Global prevalence of chronic kidney disease – a systematic review and meta-analysis. Plos One. 2016;11:e0158765. doi:10.1371/journal.pone.0158765 27383068 PMC4934905

[18] Lea JP, Nicholas SB Diabetes mellitus and hypertension: key risk factors for kidney disease. J Natl Med Assoc. 2002;94:7S–15S. 12152917 PMC2594170

[19] McClellan WM, Flanders WD Risk factors for progressive chronic kidney disease. J Am Soc Nephrol. 2003;14:S65–S70. 10.1097/01.ASN.0000070147.10399.9E 12819305

[20] Chang A, Kramer H CKD progression: a risky business. Nephrol Dial Transplant. 2012;27:2607–2609. 10.1093/ndt/gfs095 22555251

[21] Bleyer AJ, Shemanski LR, et al Tobacco, hypertension, and vascular disease: risk factors for renal functional decline in an older population. Kidney Int. 2000;57:2072–2079. 10.1046/j.1523-1755.2000.00056.x 10792626

[22] Davidman M, Olson P, Kohen J, et al Iatrogenic renal disease. Arch Intern Med. 1991;151:1809–1812. 1888247

[23] Moynihan R, Glassock R, Doust J Chronic kidney disease controversy: how expanding definitions are unnecessarily labelling many people as diseased. BMJ. 2013;347:f4298 10.1136/bmj.f4298 23900313

[24] Gallagher H, Methven S, Casula A, et al A programme to spread eGFR graph surveillance for the early identification, support and treatment of people with progressive chronic kidney disease (ASSIST-CKD): protocol for the stepped wedge implementation and evaluation of an intervention to reduce late presentation for renal replacement therapy. BMC Nephrol. 2017;18:131 10.1186/s12882-017-0522-9 28399810 PMC5387350

[25] Brock D, Abu-Rish E, Chiu C, et al Interprofessional education in team communication: working together to improve patient safety. BMJ Qual Saf. 2013;22:414–423. 10.1136/bmjqs-2012-000952 23293118

[26] Imison C, Curry N, Holder H, et al Shifting the balance of care: great expectations. [Online] 2017 March [cited 2017 May 8]. Available from: https://www.nuffieldtrust.org.uk/files/2017-02/shifting-the-balance-of-care-summary-web-final.pdf

[27] Iseki K Factors influencing the development of end-stage renal disease. Clin Exp Nephrol. 2005;9:5–14. 10.1007/s10157-005-0341-3 15830267

[28] Chronic obstructive pulmonary disease (COPD) statistics. *British lung foundation**.* [Online]. [cited 2017 May 18]. Available from: https://statistics.blf.org.uk/copd

[29] Anderson S, Halter Jeffrey B, William R, et al Prediction, progression, and outcomes of chronic kidney disease in older adults. JASN. 2009;20:1199–1209. 10.1681/ASN.2008080860 19470680

[30] Montgomery-Taylor S, Watson M, Klaber R Child health general practice hubs: a service evaluation. Arch Dis Child. 2016;101:333–337. 10.1136/archdischild-2015-308910 26699536

[31] Institute of Medicine committee on quality of health care in America. Crossing the quality chasm: a new health system for the 21st century. *The National Academies Press**.* [Online] 2001 [cited 2017 May 18]. Available from: https://www.nap.edu/read/10027/chapter/1#xiii

[32] Bridges DR, Davidson RA, Odegard PS, et al Interprofessional collaboration: three best practice models of interprofessional education. Med Edu Online. 2011;16: doi:10.3402/meo.v16i0.6035 PMC3081249 21519399

[33] A new relationship with people and communities: The report from the People and Communities Board to the Chief Executive of NHS England. *National voices**.* [Online] 2017 February [cited 2017 May 18]. Available from: https://www.nationalvoices.org.uk/sites/default/files/public/publications/a_new_relationship_with_people_and_communities_0.pdf

[34] A new relationship with people and communities: The report from the People and Communities Board to the Chief Executive of NHS England. *National archives HM Treasury**.* [Online] 2002 April [cited 2017 May 18]. Available from: https://webarchive.nationalarchives.gov.uk/+/https:/www.hm-treasury.gov.uk/consult_wanless_final.htm

[35] New care models. *NHS England**.* [Online]. [cited 2017 May 18]. Available from: https://www.england.nhs.uk/ourwork/new-care-models/

[36] How hospital activity in the NHS in England has changed over time. *The King`s Fund**.* [Online] 2016 December [cited 2017 May 18]. Available from: https://www.kingsfund.org.uk/publications/hospital-activity-funding-changes

